# *Chickspress:* a resource for chicken gene expression

**DOI:** 10.1093/database/baz058

**Published:** 2019-06-10

**Authors:** Fiona M McCarthy, Ken Pendarvis, Amanda M Cooksey, Cathy R Gresham, Matt Bomhoff, Sean Davey, Eric Lyons, Tad S Sonstegard, Susan M Bridges, Shane C Burgess

**Affiliations:** 1 School of Animal and Comparative Biomedical Sciences, University of Arizona, Tucson AZ, USA; 2 Institute of Genomics, Biocomputing & Biotechnology, Mississippi State University, Starkville MS, USA; 3 School of Plant Sciences, CyVerse, University of Arizona, Tucson AZ , USA; 4 United States Department of Agriculture Agricultural Research Service Beltsville Agricultural Research Center, Beltsville MD, USA; 5 Department of Computer Science and Engineering, Mississippi State University, Starkville MS, USA

## Abstract

High-throughput sequencing and proteomics technologies are markedly increasing the amount of RNA and peptide data that are available to researchers, which are typically made publicly available via data repositories such as the NCBI Sequence Read Archive and proteome archives, respectively. These data sets contain valuable information about when and where gene products are expressed, but this information is not readily obtainable from archived data sets. Here we report Chickspress (http://geneatlas.arl.arizona.edu), the first publicly available gene expression resource for chicken tissues. Since there is no single source of chicken gene models, Chickspress incorporates both NCBI and Ensembl gene models and links these gene sets with experimental gene expression data and QTL information. By linking gene models from both NCBI and Ensembl gene prediction pipelines, researchers can, for the first time, easily compare gene models from each of these prediction workflows to available experimental data for these products. We use Chickspress data to show the differences between these gene annotation pipelines. Chickspress also provides rapid search, visualization and download capacity for chicken gene sets based upon tissue type, developmental stage and experiment type. This first Chickspress release contains 161 gene expression data sets, including expression of mRNAs, miRNAs, proteins and peptides. We provide several examples demonstrating how researchers may use this resource.

## Introduction

Chicken is a key production animal and an important experimental model used to study developmental biology, immunology, virology and oncogenesis (1). Red Jungle Fowl is the progenitor of domesticated chickens, and the archetypical chicken genome sequence is obtained from an inbred Red Jungle Fowl female (2). This chicken genome has since served as the evolutionary linker between mammals and other vertebrates. Since its release in 2005, there have been multiple updates (2–4) to this first published chicken genome sequence, incorporating both new sequence data and improved structural annotation. Chicken is one of the species that is the focus of an ENCODE project to identify functional elements within animal genomes (5)—a project that is expected to substantially increase available chicken gene expression information.

The availability of the chicken genome also served as a resource for comparative and functional genomics projects, including almost 10 000 expression data sets in the NCBI Gene Expression Omnibus (GEO) (6) and ArrayExpress (7) archives. However, submission to these resources typically under-represents the true number of expression data sets; not all expression data is handled by GEO and ArrayExpress (e.g. proteomics data): some data sets are withheld pending publication, others are published without explicit GEO links, and still more are never submitted to a public repository. This loss is caused by incomplete data sharing, which is compounded when researchers are unable to effectively and rapidly access shared gene expression data to inform their own research.

Sequence repositories and archives are designed to promote data sharing and re-use and are not inherently useful for querying tissue expression patterns and identifying gene sets expressed in specific tissues. Several resources provide value-added gene expression resources (8); however, these contain little or no chicken gene expression information. For example, the EBI Gene Expression Atlas incorporates transcript expression data (9) but currently contains only 20 chicken experiments. While some model organism databases provide extrapolated expression information, there is no model organism database available for chicken (or any other bird species) and resources for avian species are fragmented. The rapid accumulation of chicken expression data—and the information that this data can provide in support of understanding gene function, anatomy and phenotype—underscore the need for, and utility of, a value-added gene expression resource for agriculture and biomedical research.

We report the development and deployment of Chickspress, a resource for querying chicken expression data. Chickspress includes a core set of Red Jungle Fowl RNA and peptide data collected across multiple tissues from both male and female birds and public expression data sets (3). This report describes products identified from the core data set and highlights how this coordinated data can be utilized for a better understanding of gene expression. Each experiment is linked to experimental data that includes tissue and experimental type so that researchers may easily search for information about a single gene of interest or download data about gene expression across tissue types. Since there is no model organism database for chicken, gene models provided by both NCBI (10) and Ensembl (11) are commonly used by the chicken research community, and Chickspress includes both gene sets for easy comparison and identification of genes. Expression information can also be viewed on a genome browser and trait information is included (12) to link gene expression to phenotype.

## Methods

### Sample collection

Tissue samples were collected from individuals of the same inbred UCD001 line of Red Jungle Fowl used to prepare linkage maps and for genome sequencing (13,14). Adult birds (female and male) 2.5 years old were killed and multiple tissues collected and snap-frozen in liquid nitrogen.

### Transcriptome sequencing

Total RNA was extracted from each tissue with the MiRNeasy kit (Qiagen, Valencia, CA, USA) according to the standard protocol. Total RNA quality was determined using a Bioanalyzer 2100 (Agilent, Santa Clara, CA, USA). A paired-end Illumina mRNA-Seq library was built for each tissue sample using the TruSeq RNA Sample Preparation kit (Illumina, San Diego, CA, USA) according to the standard protocol. A single-end Illumina small RNA-Seq library was built for each tissue sample using the TruSeq Small RNA Sample Preparation kit (Illumina, San Diego, CA, USA). All Illumina libraries were quantified using the standard Illumina qPCR quantification protocol. Indexed samples were multiplexed at a concentration of 1 pM each in sets of four and clustered on an Illumina cBot. Paired-end sequencing of mRNA libraries was performed for 200 cycles and single-read sequencing of small RNA libraries was performed for 50 cycles on an Illumina HiSeq 2000 instrument using TruSeq SBS chemistry v3 (manufacturer’s protocols). Base calling was done on the instrument by Illumina RTA 1.13.48. De-multiplexing and conversion of per-cycle base call files (*.bcl) to FASTQ files was done using CASAVA 1.8.2. De-multiplexing criteria allowed for one mismatch, and the resulting FASTQ files contained Sanger format ASCII encoded quality scores.

### mRNA analysis

All reads were quality trimmed and adapters were removed using Trimmomatic 0.25 (15). Adapters were removed from paired end reads using the ILLUMINACLIP function. Two mismatches were allowed between the adapter/primer sequence and the read. A Q30 score was required in order to remove read-through sequences, and a Q15 score was required to remove adapter/primer sequences from the end of the read. Bases with quality scores below 30 were trimmed from both ends of the reads. Only trimmed reads with a minimum length of 30 bases were kept. Single-read small RNA data were trimmed in the same manner but were kept if longer than 18 bases. All paired-end mRNA reads were mapped to the chicken genome (Galgal5; for more details, see Chickspress section, below) using Tophat (v 2.1.1) (16) with the Bowtie2 (v 2.2.9) algorithm (17). The insert size was 170 bases with a mate standard deviation of 70 bases, minimum intron size of 25 bases, microexon search and the -g option with supplied annotations in GFF3 format (NCBI 103 or Ensembl 89). Data for each sample was mapped twice with Tophat—once with NCBI annotations supplied and once with Ensembl annotations supplied.

Transcripts were assembled and their abundances estimated using Cufflinks (v2.2.1) (18) with the -g option to use known annotations as a guide while still generating novel transcripts. Cufflinks analyses were done using both NCBI and Ensembl annotations. The maximum bundle length was increased to 9 700 000 bases to allow for spliced mappings to non-adjacent contigs present on the artificial chromosomes. This change was made as the result of skipped bundles seen on the chrUn sequence with the default maximum bundle size parameter. No skipped bundles were found on other chromosomes. The resulting GTF files were combined using the Cuffcompare module of the Cufflinks program incorporating names and accessions from both NCBI and Ensembl where appropriate. The Cuffcompare -C option was used such that all transcripts were recorded in the output file. A final GFF3 file was created for each sample for display on Chickspress and is available for download. All novel mRNA transcripts were assigned `CXT’ identifiers.

### Small RNA analysis

All reads were mapped to the chicken genome with Bowtie2 (v2.2.9) (17) using fast, end-to-end mode. The resulting SAM files were converted to GFF files for use with the miRTrap software (19). miRTrap was used to predict both mature and primary miRNAs based on read region length, RNA folding, average anti-sense product displacement and non-miR neighbor count. Appropriate miRBase names and accessions were transferred to any predicted miRNA for which the coordinates were considered the same as a known miR (+/−5 nucleotides for mature miRNA or with any overlap of a known primary miRNA transcript). NGSUtils (v0.5.2b) was used to calculate FPKM values for all small RNA (20). Targets for miRNAs were predicted using miRanda (v3.3a) with energy −20 and score 155 cutoffs.

### Proteomic analysis

For the proteomic analysis, 100 mg of each tissue was subject to differential detergent fractionation and 20 μg of each fraction was trypsin digested as previously described (21, 22). Following digestion, each fraction was desalted using a peptide macrotrap (Michrom BioResources) according to the manufacturer’s instructions. After desalting, each fraction was further cleaned using a strong cation exchange macrotrap (Michrom BioResources) to remove any residual detergent, dried and resuspended in 10 μl of 2% acetonitrile and 0.1% formic acid and transferred to low-retention vials in preparation for separation using reverse-phase liquid chromatography.

An Ultimate 3000 (Dionex) high-performance liquid chromatography system coupled with an LTQ Velos Pro (Thermo) mass spectrometer were used for peptide separation and mass spectrum acquisition. The U3000 was operated at a flow rate of 333 nl per minute and equipped with a 75 μm × 10 cm fused silica column packed with Halo C18 reverse phase material (Mac-Mod Analytical). Each peptide sample was separated using a 4-h gradient from 2 to 50% acetonitrile with 0.1% formic acid as a proton source. The column was located on a Nanospray Flex Ion Source (Thermo) and connected directly to a stainless steel nanospray emitter to minimize peak broadening. Scan parameters for the LTQ Velos Pro were 1 MS scan followed by 20 MS/MS scans of the 20 most intense peaks, all collected in normal scan mode. High-energy collisional dissociation was chosen as the fragmentation method. Dynamic exclusion was enabled with a mass exclusion time of 3 min and a repeat count of 1 within 30 sec of initial m/z measurement.

Spectrum matching programs X!tandem (23) were used via the University of Arizona High-Throughput Computing Center to search mass spectra against the NCBI RefSeq and Ensembl protein sets for *Gallus gallus*. RefSeq proteins were bundled in FASTA format with release 103 of the NCBI chicken genome annotation and Ensembl proteins accompanied with release 89 of the Ensembl annotations. Raw spectra were converted to mgf format for analysis using the MSConvert program from the ProteoWizard software suite (24, 25). X!tandem was run with 12 threads, precursor and fragment tolerance of 0.5 Da, and up to two missed tryptic cleavages. A custom Perl script was used to parse XML search results from X!tandem and organize them by tissue. Peptides with *E*-values of ≤0.01 were accepted and single-spectrum identifications were rejected unless they were identified by both search engines. To verify data set quality, decoy searches were done exactly as above, but using randomized versions of the RefSeq and Ensembl protein databases. False discovery rates ranged from 0.00 to 0.85% with an average of 0.22%.

### Proteogenomic analysis

Proteogenomic mapping requires mass spectra to be matched against the six-frame translation of a genomic sequence (21, 26). Because the chicken genome is ~1 Gb in size, computer memory requirements are larger than the available hardware allows. Also, X!tandem calculates *E*-values for spectrum matches based on the size of the database used, as the *E*-value by definition is the frequency of obtaining a score at least as good as the best match for a spectrum in a database of a particular size. Genome-sized databases introduce large amounts of noise, and the scoring algorithm of X!tandem assigns *E*-values, which are low in confidence. To address these issues, a custom Perl script was used to process the genome sequence and divide it into smaller segments. Each chromosome was translated into six frames using the standard genetic code. Next, the amino acid sequence for each frame was split into segments 600 amino acids long, with a 60-amino-acid overlap in between segments so that peptides that might span segments can be identified. These segments were then grouped into FASTA files of 50 000 segments each. The result is a group of 69 FASTA files (~34 Mbytes each) containing 50 000 entries, representing the entire six-frame translation of the chicken genome. Because the memory requirements and entry size are more similar to a standard protein database, X!tandem is easily able to process these files, and the *E*-value calculations are not affected by excess noise. One of the FASTA files was randomized for decoy searches and false discovery rate calculations. Genome searches were done identically to the protein searches. Spectrum matches were accepted for *E*-values of ≤0.01, and single-spectrum identifications were removed. Because the same spectra were searched against multiple FASTA files, if more than one peptide sequence was matched per spectrum, only the match with the best *E*-value was accepted. False discovery rates ranged from 0.00 to 0.71% with an average of 0.35%. Prior to peptide mapping, proteome and genome search results were combined by tissue. As with the genome results, if a proteome spectrum was matched to multiple peptide sequences, the match with the lowest *E*-value was accepted. Identified peptide sequences were matched against the chicken genome sequence using a custom Perl script.

### Developing the Chickspress resource

Chickspress was built using the CoGe genome browser (27). The reference information provided to the genome browser were the NCBI *G. gallus* 5.0/galGal5 assembly (Dec 2015; ICGSC), NCBI gene models (annotation release 103; January 2016), Ensembl gene model (release 89; Dec 2016). This genome browser currently includes 39 mRNA experiments, 77 miRNA experiments and 29 proteomics experiments. The Chickspress search and download data application runs on a Linux server using a MySQL database and a Perl CGI application. It searches the experimental chicken data using experimental metadata and sequence features to generate data that can be downloaded in several different formats.

Chicken QTL tracks are loaded from the Animal QTLDB (12) release animalQTL_rel32 Apr2017 GFF3 files. For each QTL GFF3, we generate new GFF3 files by mapping traits in these files to their corresponding ontologies (VT, CMO, PTO) and generating the following trait types: behavior, biochemistry/metabolism, conformation, disease susceptibility, egg production, egg quality, fatness, feeding, growth, health (other), meat quality, pigmentation association and production other). These trait types are consistent with the current Animal Genome QTLdb JBrowse traits, allowing researchers to identify traits between these two resources.

### Data accessibility

All core data sets produced as part of this project are freely and publicly available via the Chickspress resource and are submitted to the public data archives. RNA-Seq data (obtained from both mRNA and small RNA libraries) is available at the NCBI SRA, listed under NCBI BioProject PRJNA204941. Proteomics data obtained from the same Red Jungle Fowl tissues is available from the Massive repository, accession MSV000080320, and the ProteomeXchange repository, accession PXD005288. In addition, experimental tracks from Chickspress can be directly downloaded from the CoGe browser by selecting Experiment View from the right-hand side track selection pane, selecting Experiment View and selecting Download from the Export Data option on the top left-hand side. We also accept expression data from the research community and these data sets are listed with the Principal Investigator’s name; this data remains the property of the submitter, and where it is available, we provide links to public archive and publication details for this data.

## Results

This project is unique in that we measured gene expression in tissues from individuals of the same inbred genetic line as the individual used to develop the reference genome; further, we measured mRNA, miRNA and protein expression from the same sample for multiple tissues to make this survey more comprehensive. This core set includes 13 tissues from a female adult bird and the corresponding tissues from a male bird (Table 1).

**Table 1:** Summary of the core expression data that forms the basis of the Chickspress resource

**Sample**		**Transcripts**		**Proteins/Peptides**	
**Tissue**	**Sex**	**mRNA**	**Mature miRNA**	**Annotated proteins**	**Novel peptides**
Adipose	Female	59 813	2632	5214	8046
	Male	59 153	1200	797	496
Cerebellum	Female	70 332	1738	7043	16 235
	Male	69 673	2588	10 384	21 218
Cerebrum	Female	62 656	1134	5008	9977
	Male	62 696	1590	1928	1711
Gonad	Female	114 221	1272	4980	8133
	Male	122 409	2004	2427	3406
Heart	Female	62 981	1117	4532	10 915
	Male	89 123	439	6191	18 714
Hypothalamus	Female	72 147	1473	9418	15 030
	Male	72 557	1567	12 977	26 499
Kidney	Female	71 254	902	5224	6582
	Male	55 938	992	5258	14 493
Liver	Female	49 867	958	5770	15 548
	Male	57 268	1330	6845	26 400
Lung	Female	115 033	1345	5205	12 409
	Male	73 416	1173	5204	18 738
Muscle (breast)	Female	47 396	1474	4156	16 523
	Male	43 643	988	3579	6315
Nerve (sciatic)	Female	61 030	1092	8662	7357
	Male	72 713	1423	3478	7737
Proventriculus	Female	48 271	823	4744	9787
	Male	46 921	95	7646	17 287
Spleen	Female	173 488	1022	4876	12 943
	Male	168 215	970	7916	24 279

All samples are from adult Red Jungle Fowl tissues, the same population that was sequenced to determine the chicken genome.

Annotation to identify genes and gene products is an ongoing process, and we used data generated by this core set of experiments to identify expression previously unannotated gene products (referred to as `novel’). Comparison of the core set to both NCBI (10) and Ensembl (11) gene models identified 985 745 novel transcripts, 14 692 novel miRNAs and 245 927 novel peptides expressed across these core tissues. We note that 98% of these novel elements are identified from single tissues. Many of these novel expression products represent corrections to existing gene models, demonstrating limitations of the current computational annotation pipelines used for all genomes (discussed below).

While new sequencing technologies mean that the amount of tissue expression information is rapidly accumulating for a broad range of species, existing gene expression resources to access and view this information are either limited to a single species and/or limited expression data types. In addition to providing information about tissue- and sex-specific expression, collecting information about mRNA, ncRNA and protein expression from the same sample provides information about transcriptional and translational regulation processes (e.g. relationships between regulatory ncRNAs and mRNAs and between mRNAs and protein isoforms). Likewise, we anticipate that the addition of gene expression information from different developmental stages to support the identification developmentally restricted gene products and their regulation.

We also note the importance of including proteomics expression data. Proteomics information allowed us to check computational annotations for protein coding genes, better understand the effects of RNA regulation upon protein expression and investigate how the expression of mRNA (routinely used us a gene expression proxy for protein coding genes) corresponds to protein expression. Finally, we make all of the core information for the Chickspress gene expression data sets publicly available, ensuring that this data is used for improvements in future genome annotation. The release of improved chicken genome sequence and annotation will improve the accuracy of all chicken `omics-based’ studies (5) and improve our understanding of avian genomes (4, 28), and corrections to annotation pipelines will have wide ranging implications for all species.

### mRNA identification

**Figure 1:**
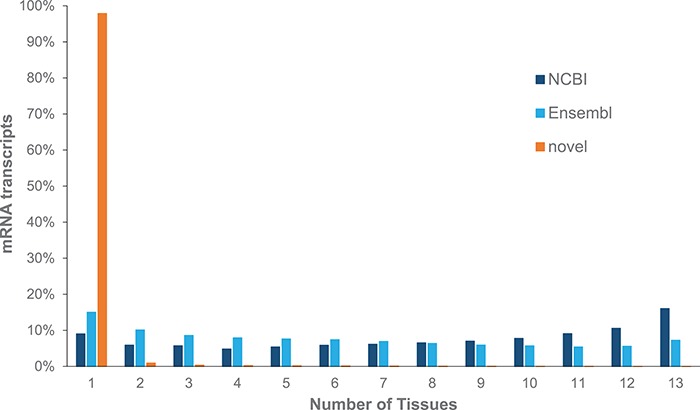
Expression of mRNAs across multiple tissues from adult Red Jungle Fowl. mRNA transcripts are grouped based upon whether they are annotated by NCBI or Ensembl or if they are novel.

We identified 985 745 transcripts from our core set of tissues; only 7.9% were found to be previously annotated by NCBI or Ensembl gene sets, while 3.35% were identified from both NCBI and Ensembl gene annotation pipelines. We note that 97.9% of these novel transcripts are identified from single tissues, while most mRNAs from NCBI and Ensembl are identified in multiple tissues (Figure 1). These novel mRNAs represent a mixture of unannotated isoforms, transcripts found within an annotated intron and intergenic transcripts (Supplementary Table 1), with the latter two classes likely to represent previously unannotated ncRNAs.

### miRNAs identification

**Figure 2:**
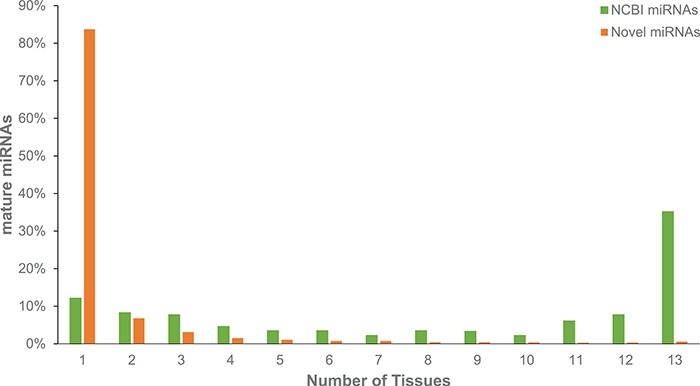
Expression of miRNAs across multiple tissues from adult Red Jungle Fowl. Mature miRNAs are grouped based upon whether they have been annotated in NCBI or if they are novel and are reported based upon the number of tissues in which they were identified.

Across all 26 Red Jungle Fowl tissues we identified 14 a692 mature miRNAs, including 543 previously identified and available from NCBI Entrez (10). This number represents 72.5% of chicken miRNAs identified by NCBI; however, there are many reported chicken miRNAs that are not in public resources, and this number likely under-represented the true number of `known’ miRNAs in chicken. Similar to the identification of novel mRNA transcripts, novel miRNAs are predominantly identified from single tissues (Figure 2). Additional miRNA information is provided by Dr HC Liu North Carolina State University (NCSU), who surveyed adipose, Bursa, liver, spleen and thymus tissues from three embryological stages and seven post-hatch stages (3). A comparison of miRNAs predicted from these immunological tissues indicates that only 472 of our novel miRNAs were also found in these tissues; this is perhaps not surprising given the role of miRNAs in gene regulation. This finding reiterates the importance of surveying a broad range of tissue types and developmental stages to identify elements involved in gene regulation. Chickspress, by providing a unifying repository for the community (including yet to be published data), will facilitate more accurate modeling of these regulatory elements.

### Protein identification

**Figure 3:**
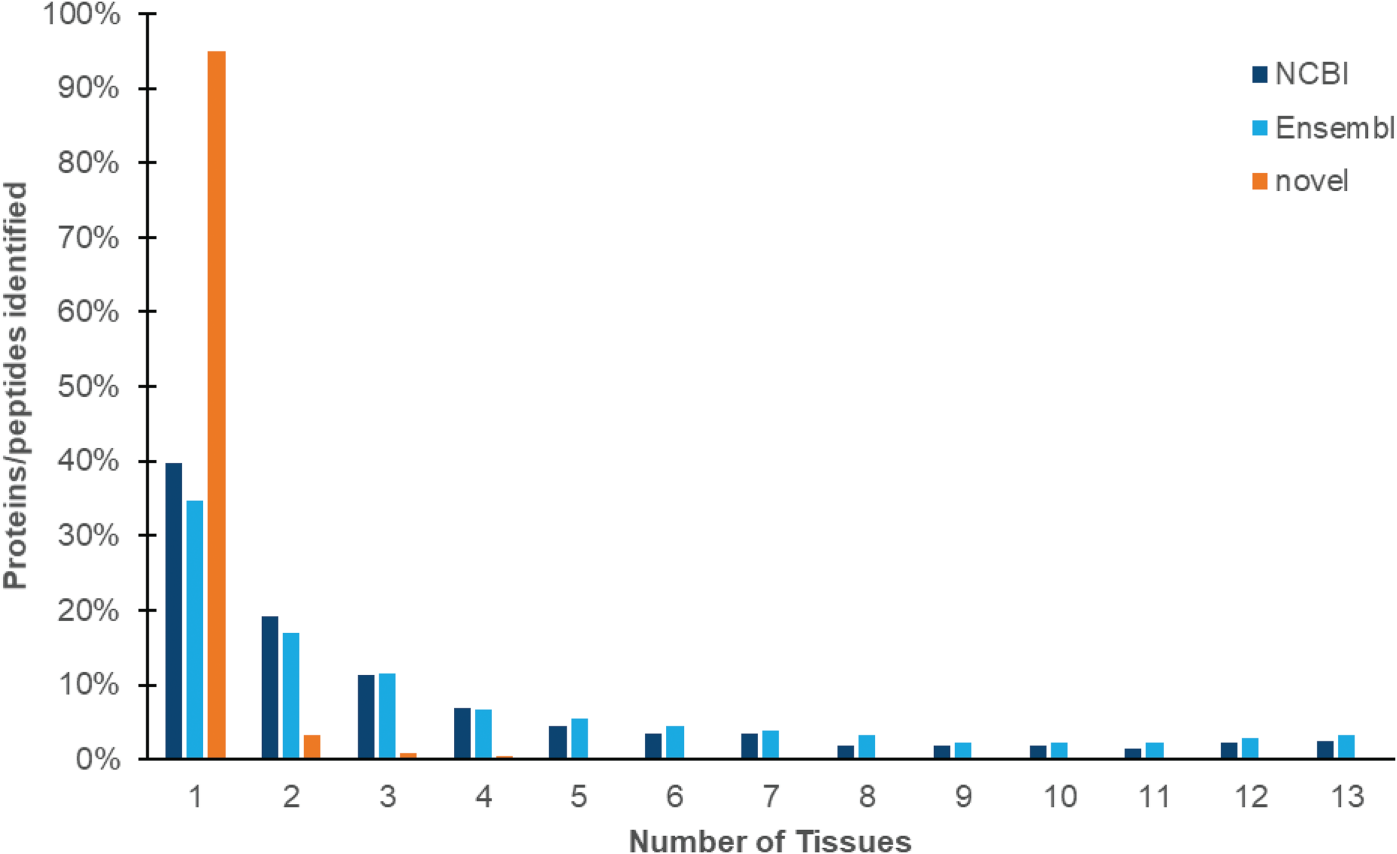
Expression of proteins and peptides across multiple tissues from adult Red Jungle Fowl. Peptides are identified as mapping to NCBI and Ensembl proteins, or as novel proteogenomic peptides (which map to the genome but not to annotated proteins). All peptides included in this study have a *P*-value of ≤0.01.

We identified a total 187 000 peptides (Eval, ≤0.01; peptide false discovery rate of 0.71%). Of these, 33 679 peptides (18.0%) map to 30 079 proteins from NCBI and Ensembl proteins. The remainder of these peptides is mapped to regions of the genome that are currently characterized as non-coding, and we designate these proteogenomic peptides as `novel’. Like mRNA transcripts, both annotated proteins and novel peptides are predominantly identified only once from the core experimental data set (Figure 3).

The identification of proteogenomic peptides has been reported in studies with diverse species, from animals to DNA viruses (21, 29, 30). Initially, these peptides were considered to be an artifact of proteomic discovery; however, improvements to proteomic software that include correcting for false discovery and assigning *E*-values to peptide identification have not eliminated their occurrence. This process is further complicated because proteogenomic peptides may map to more than one genomic location. Although the chicken genome has less repetitive sequence than mammalian genomes (13), the number of `novel’ or proteogenomic peptides that map to a single genomic location is 134 216 peptides, or 141 times the false discovery rate for this data set. By comparing the genomic location of these novel peptides to gene models from NCBI and Ensembl we classified novel peptides that map to a single genomic location relative to current annotation of genes (Table 2). Most novel peptides map to intergenic regions and may represent small ORFs (<100 aa) that annotation pipelines discard. Moreover, nearly all peptides that map to 5′- and 3′UTR regions occur in the Ensembl annotations, while 5′- and 3′-exon extensions predominantly occur in NCBI annotations, suggesting biases in these pipelines.

**Table 2:** The classification of novel peptides in relation to annotated genes

**Peptide category**	**Mapped in NCBI & Ensembl**	**NCBI peptides**	**Ensembl peptides**	**Total peptides**
Intergenic	87 208 (81.6)	3949 (3.7)	15 735 (14.7)	106 892
Intron	17 823 (59.0)	8844 (29.3)	3563 (11.8)	30 230
Transcript (noncoding)	83 (1.5)	5255 (92.0)	376 (6.6)	5714
Coding out of frame	2572 (58.5)	1471 (33.5)	351 (8.0)	4394
N-ter extension	1543 (63.9)	589 (24.4)	284 (11.8)	2416
C-ter extension	1275 (66.9)	467 (24.5)	164 (8.6)	1906
5′ UTR	0 (0)	0 (0)	2058 (100.0)	2058
3′ UTR	3 (0.3)	27 (2.6)	991 (97.1)	1021
5′ exon extension	20 (1.1)	1740 (92.9)	112 (6.0)	1872
3′ exon extension	10 (1.3)	685 (92.1)	49 (6.6)	744
***Total:***	110 537	23 027	23 683	157 247

Novel peptides that mapped to a single genomic location were assigned categories based upon their relation to annotated gene models from NCBI and Ensembl, and the number of peptides in each category is reported with its proportion of the total in parentheses. Note that peptides can be classified differently between NCBI and Ensembl due to differences in annotation between these two resources. (All peptides included in this study were identified with an E-value of ≤0.01).

### Chicken gene annotation

**Figure 4:**
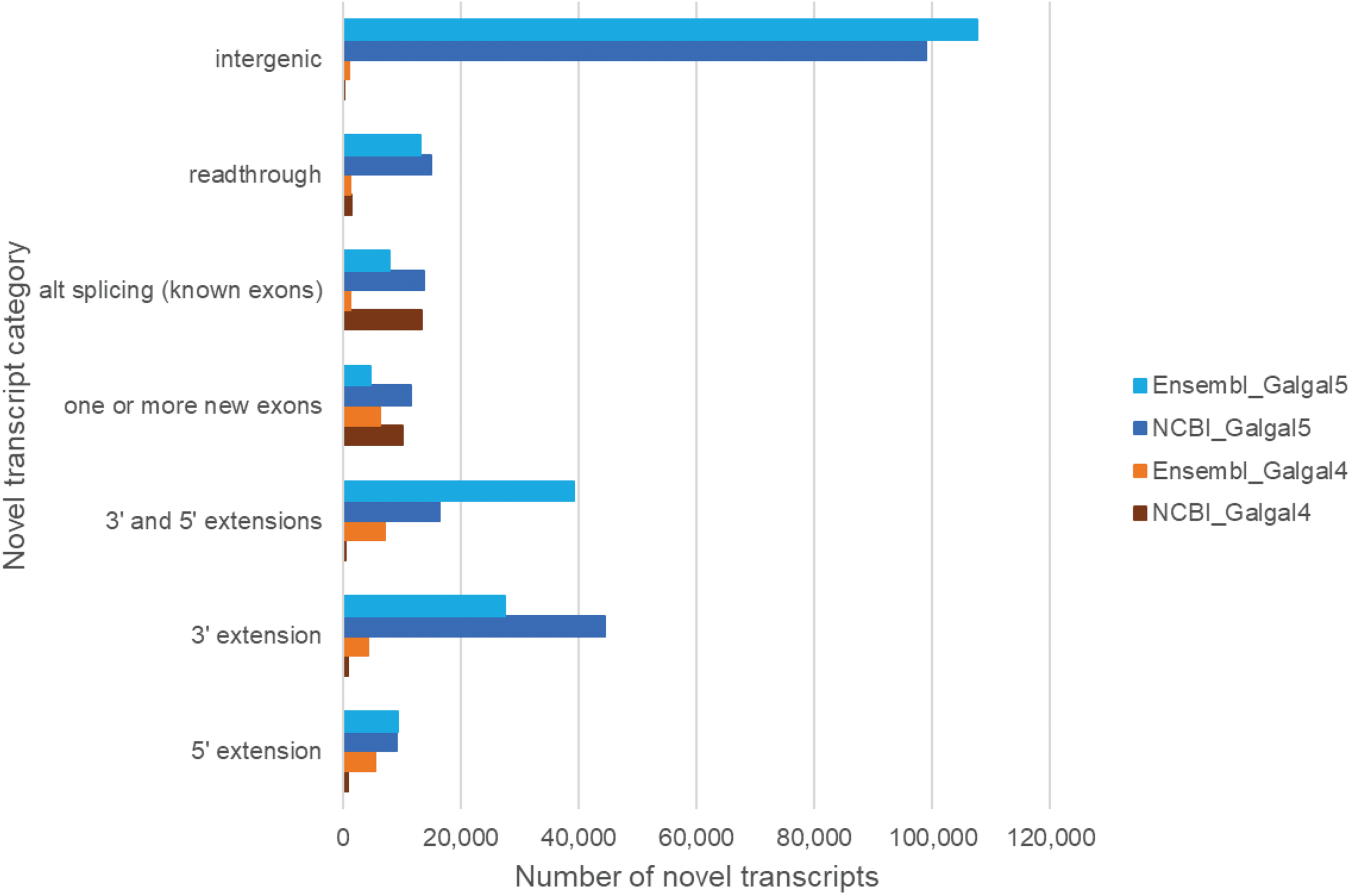
Novel transcripts not in the current chicken gene annotation. Novel transcripts are assembled mRNAs that differ from the gene model, and this difference is shown for NCBI and Ensembl genes.

Since there is no model organism resource for chicken, gene annotations from both NCBI and Ensembl are used by the community. Annotations based upon the Galgal5 assembly show a small increase in protein coding gene models identified by NCBI and Ensembl, with a 1.4–2-fold increase in mRNA transcripts and the addition of non-coding RNA genes (Table 3). Our mapping to Galgal5 gene models resulted in a 9-fold increase in novel transcripts (denoted with a CXT prefix), compared to Galgal4 gene models. To investigate this further, coordinates for novel transcripts were compared to NCBI and Ensembl gene models and grouped based upon their differences to these models (Figure 4). Our results show that Galgal5 gene annotations from both NCBI and Ensembl are missing 5′ and 3′-termini but also that most novel transcripts are located in regions where there is currently no gene annotation (`intergenic’). Further inspection of these novel `intergenic’ transcripts indicates that they represent smaller transcripts than the annotated RNAs. While annotated mRNAs have an average of 12.8 exons per transcript, the CXTs have an average of 6 exons. Moreover, the chicken genome contains multiple unplaced scaffolds, the CXTs identified from scaffold sequence have an average of 3.5 exons/transcript. Given that most CXTs are identified from a single tissue (Figure 1) and are smaller than annotated mRNAs, it is not surprising that these novel transcripts are missed or discarded by current annotation pipelines. Our work also re-emphasizes the limitations of relying on *in silico* only models, and provides information to inform annotation pipelines.

**Table 3:** Chicken gene annotation

**Gene models**	**Ensembl**		**NCBI**	
	***Galgal4***	***Galgal5***	***Galgal4***	***Galgal5***
**Protein coding genes**	17 108	18 367	21 331	26 640
mRNAs	16 354	30 252	32 189	46 389
**ncRNA**			498	9231
lincRNA		5972		
miRNA		1116		
rRNA		203	3	3
tRNA			311	312
snoRNA		233		
snRNA		112		
**Unassigned transcripts**	1600	187	4375	2156
**Pseudogenes**	0	86	304	0

Gene annotation based upon the Galgal5 assembly shows a small increase in gene model numbers for both NCBI and Ensembl annotations. In contrast, new sequence data types allowed the identification of noncoding RNAs previously not annotated by NCBI or Ensembl

### Usage and utility

We provide matched, quantitative gene expression data that allows researchers to directly compare RNA and protein expression levels from female and male tissues. This matched expression data is calculated across multiple tissues from the same bird (for both female and male) so that researchers can compare gene expression in multiple tissues. These matched data sets also provide information for researchers who use mRNA as a proxy for protein expression *in vivo* and is likely to provide insight into the regulation of gene expression through translational control and protein turnover. We also display experimental data sets collected by other researchers. This provides additional expression information data that spans different tissues, ages and lines.

The online Chickspress resource is provided as a genome browser or, for more complex searches involving gene sets, there is the `Chickspress Search and Download Data’ option at the top of the page. The genome browser allows researchers to rapidly identify corrections to existing genomic elements and novel elements and tissue specific expression for particular genes. We have also added chicken QTL and trait information from the Animal QTLDB so that researchers may view these genomic regions and determine genes expressed in tissues relevant to these traits. In addition to viewing individual genes or genomic regions on a genome browser, researchers may also choose to data mine this information. Individual experiments are listed by investigator name, tissue, sex and experiment type (e.g. transcript, proteomics), and each of these features are searchable. Additional information that we collect for each experimental data set includes public repository accession, associated publication, genome version, breed or line, age or developmental stage and disease status. For transcript experiments we also collect information about read type (e.g. single or paired end), average insert and read size, number of input reads, flow cell ID, alignment software and analysis software. Where possible, ontologies and controlled vocabularies are used to avoid free text and promote searching capabilities, for example tissue types are described using the BRENDA tissue ontology terms (31). The examples below provide case studies for how researchers may use Chickspress.

### Simple gene searches

**Figure 5:**
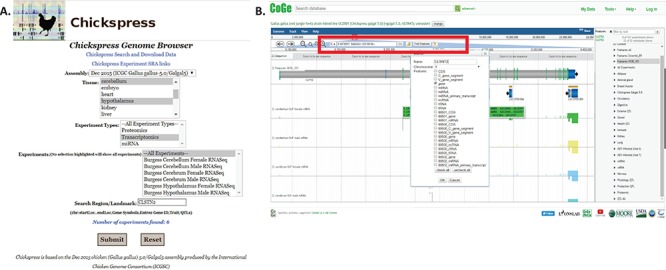
Searching for specific genes in Chickspress. (A) Searching for CLSNT2 expression in nerve tissues using the Chickspress Genome Browser main page. (B) Researchers may also directly search from the CoGe browser by entering a region or using the `Find Features’ option (red box). Identified features are displayed in the right-hand side menu.

A typical query would be to investigate the expression of a single gene across multiple tissues. From the Chickspress Genome Browser page, researchers may select tissues and experiment types and search for different regions by location or landmarks using gene names or accessions (Figure 5A). From the CoGe genome browser, researchers may select to view a specific region by entering genomic location, accessions or gene symbols at the top of the browser screen (Figure 5B). However, we strongly recommend that researchers use accessions if at all possible; chicken gene nomenclature is a work in progress and very often different gene names and symbols are assigned to the same genes from NCBI and Ensembl.

**Figure 6:**
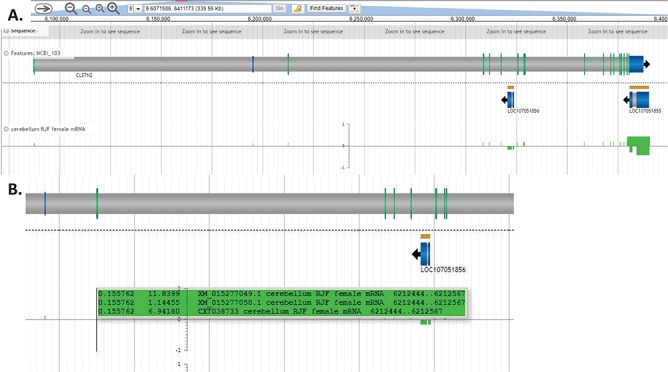
Expression of individual transcripts in the CoGe browser. (A) Individual experiments are displayed as separate tracks, with genes in the forward orientation represented as positive value and genes in the reverse orientation represented as negative value. (B) Holding the cursor over exons displays individual transcripts and their expression values. The first value is exon expression, and the second value is expression of the full-length transcript (all expression values are calculated as FPKM). This view may be regenerated at https://goo.gl/x5vzaa.

Individual experiments are classified by lines, sex and tissue types and displayed as individual tracks (Figure 6A). The CoGe browser is fully configurable, allowing viewers to select the tracks and features they wish to be displayed. Each track shows gene expression, with auto-scaling that allows a full screen view of genomic regions. Genes in the forward orientation are shown with positive expression, and genes in the reverse orientation are shown with negative expression. To see expression values, viewers can move the cursor to the exon, enabling a pop-up screen that shows detailed transcripts and expression values (Figure 6B).

A simple gene search to identify expression patterns for a gene will show information about which transcripts (and proteins) are expressed in which tissues. For example, Calsyntenin-2 (CLSTN2) expresses proteins that regulate postsynaptic calcium concentration in neurons (32). CLSTN2 is annotated with three transcripts: XM_015277049 includes the first exon, XM_015277050 starts at the second exon and ENSGALT00000008477 starts at the fourth exon. Both XM_015277049 and XM_015277050 are more highly expressed in central nervous system tissues, while ENSGALT00000008477 has higher expression in peripheral nervous tissue. Since there is little to no evidence about specific functions of chicken transcripts, this expression information provides a basis for targeted experimental testing of these functions. Further, we note that although annotations provided by NCBI and Ensembl often differ for the same genes, these differences in transcript annotations seem to be supported by biological evidence.

**Figure 7:**
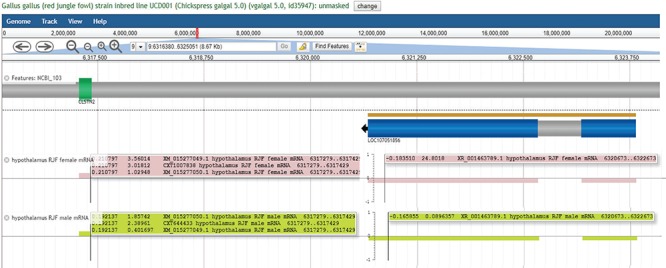
LOC107051856 expression varies in concert with CLSTN2 transcripts. LOC107051856 is an uncharacterized ncRNA nested within CLSTN2. LOC107051856 is highly expressed in female hypothalamus but not in male hypothalamus. The XM_015277049 CLSTN2 transcript is more highly expressed in female compared to male hypothalamus, while the reverse is true for the XM_015277050 CLSTN2 transcript. This view may be regenerated at https://goo.gl/x5vzaa.

In addition to identifying tissue-specific expression patterns, this type of search may also highlight sex-specific expression. For example, nested within exon 6 of CLSTN2 is LOC107051856, an uncharacterized ncRNA. This gene is most highly expressed in female hypothalamus (24.8018) but has very low expression in male hypothalamus (0.0896357). Since we did not impose any cut offs for gene expression, many gene products with very low expression values are still displayed so that researchers may determine their own relevant cut off levels. With this in mind we note that LOC107051856 expression is limited to female hypothalamus and lung (female and male), since other expression values are <0.1. LOC107051856 expression appears to coordinate with XM_015277049 and XM_015277050 expression in the hypothalamus, being highly expressed in female hypothalamus when XM_015277049 in expressed at higher levels than XM_015277050 (Figure 7). Evaluating expression patterns of protein coding genes and ncRNAs may provide clues about function of newly annotated genes.

### Search and download data sets

**Figure 8:**
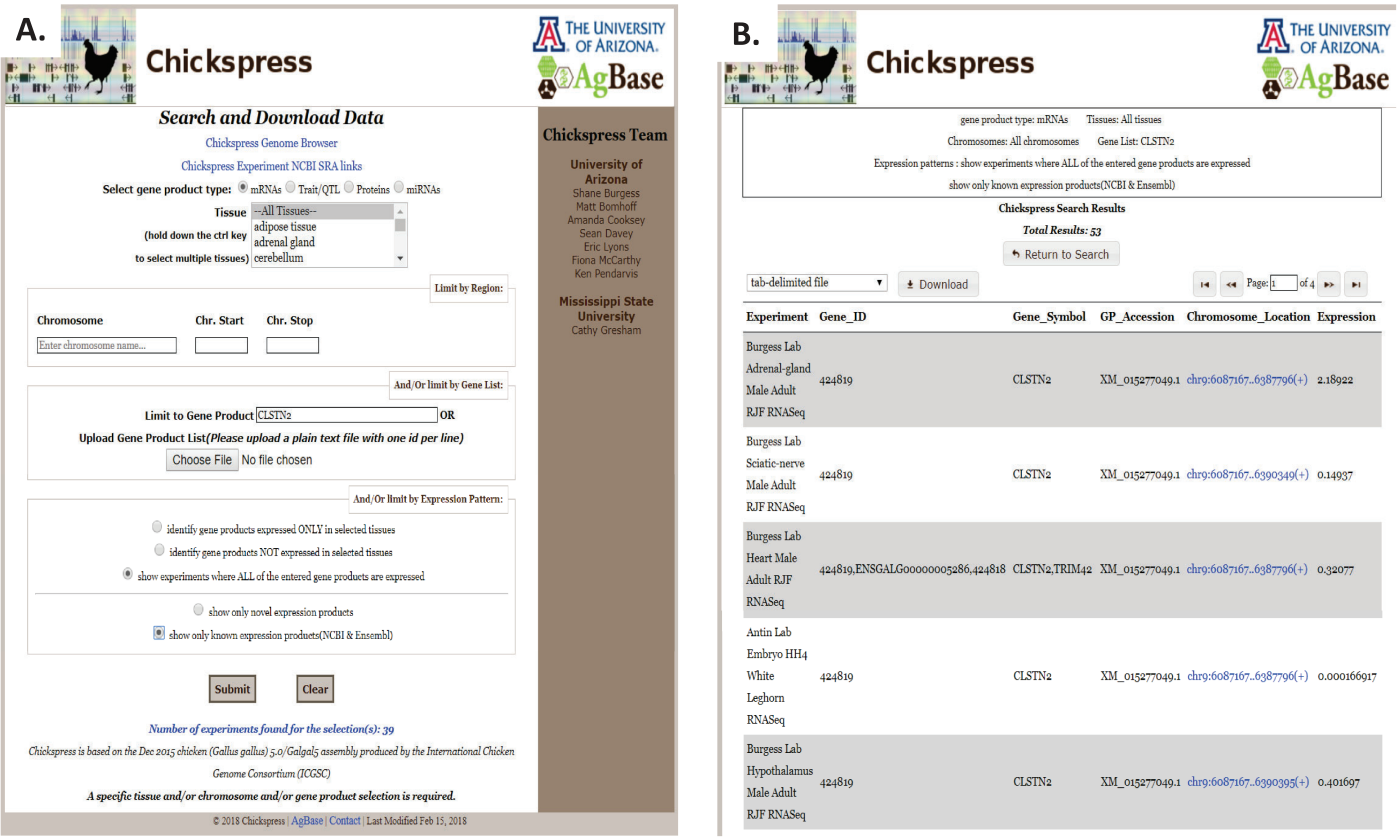
Filtering and downloading Chickspress data. (A) Chickspress data can be filtered and downloaded using the `Search and Download Data’ page. This interface allows users to filter based upon gene product type, tissue, genomic region and expression characteristics. (B) Results of a search displayed as a table with link to the genome browser. Search criteria are displayed at the top of the page, and results may be downloaded in a number of file formats.

Chickspress users may also search and download information from this resource. Selecting the `Search and Download’ link at the top of the page provides these search and download options. Information can be selected based upon gene product type, tissue or chromosomal regions, and there are options to limit by gene and expression (Figure 8A). For example, selecting all annotated mRNAs identified from the CLSTN2 gene will provide 53 results as a list of experiments with links to the genome browser (Figure 8B), and this will provide information about expression levels of CLSTN2 across different tissues. This list may be downloaded using several file format options. The tab-delimited file option downloads a file with the same information as shown in the results table and can be opened in Excel as a sortable table. The GFF format is formatted to be viewed in a genome browser, while the FASTA file format provides sequence. The GO tab-delimited file provides Gene Ontology information for the selected gene set, supporting functional analysis. For researchers who wish to investigate miRNAs, we provide the option of downloading either all predicted miRNA targets or a file that lists only predicted miRNA targets for which there is no corresponding evidence of protein expression in the same tissue. Each of these is a tab-delimited file that contains the miRNA and its expression information along with target accessions and their projected energy scores (calculated by the miRanda software).

Other types of queries that researchers may ask for include identifying gene products expressed in a specific tissue type or comparing expressed gene products between two tissue types. These types of queries, involving multiple gene products, are not well suited to display using a genome browser. We developed the capacity for researchers to search and download larger data sets, enabling them to do more complex queries of the Chickspress data. This search form allows the researcher to specify the type of gene product (transcript, protein, miRNA, QTL) and tissue(s) they wish to search. For example, a search of all novel miRNAs expressed only in chicken testes returns 1474 miRNAs. Researchers can further refine their search to include a specific genomic location (e.g. a QTL region), gene lists (e.g. all genes regulated by a certain transcription factor) and by expression pattern. For example, a researcher may wish to find all proteins identified in adipose tissue. Selecting `protein’ as the gene product type and `adipose’ as the tissue results in a list of 14 553 peptides; further refining this search by selecting `identify gene products expressed ONLY in selected tissues’ returns 8755 peptides, while adding the stipulation `show only known expression products (NCBI & Ensembl)’ identifies 498 peptides mapping to 337 protein coding genes annotated by NCBI and Ensembl.

### Future perspectives

New sequencing technologies are increasing the rate of gene expression data accumulation, and we expect the FAANG Project to also generate key data sets that relate to RNA expression and regulatory elements (5). This will provide additional information to support gene expression studies and is already contributing to the identification of ncRNA genes. Currently the Chickspress core data sets contain expression data from adult birds, and the bulk of expression data on Chickspress is also from adult birds. Chicken is an important developmental model, and, as we add more data, we expect to focus on developmental data sets. To ensure that this developmental data is consistent, searchable and comparable to other developmental models, we are also developing a chicken anatomy ontology that includes developmental terms for birds; we expect to use these ontological terms in describing the metadata of our experimental data sets.

## Supplementary Material

Table_S1_baz058

## Notes

**Database URL:** http://geneatlas.arl.arizona.edu
