# Supplementary material for: Chickspress: a resource for chicken gene expression
Source: Database (Oxford). 2019 Jun 10;2019:baz058. doi: 10.1093/database/baz058 (PMC6556980; doi:10.1093/database/baz058)
Supplement: Table_S1_baz058 [file table_s1_baz058.docx]

**Supplementary Table 1. Classification of novel transcripts identified from core data sets.** Novel transcripts are transcripts identified form the core set of Red Jungle Fowl data that have not previously been annotated by NCBI or Ensembl. Novel transcripts are classified using Cuffcompare RNA classes (the single-letter code reported by Cuffcompare is included).

| **RNA Class Description** | **Code** | **% of Novel transcripts** |
| --- | --- | --- |
| transcript contained within annotated intron | i | 31.53 |
| novel isoform | j | 30.05 |
| intergenic transcript | u | 27.77 |
| possible polymerase run-on fragment (transcript produced by transcriptional "error" | p | 5.47 |
| exon extended compared to reference (either annotation error or unspliced intron from pre-mRNA) | e | 2.95 |
| novel transcript overlaps with a reference transcript | o | 1.12 |
| novel transcript overlaps but on opposite strand to reference transcript | x | 1.07 |
| intron of the novel transcript overlaps a reference intron on the opposite strand (likely due to read mapping errors) | s | 0.04 |
